# Information-seeking on the internet during the transition to motherhood: descriptive survey, Cyprus

**DOI:** 10.1093/eurpub/ckac131.426

**Published:** 2022-10-25

**Authors:** N Middleton, I Koliandri, O Kolokotroni, E Hadjigeorgiou, V Christodoulides, C Nicolaou, C Kouta, M Karanikola, N Middleton, Baby Buddy Forward partnership

**Affiliations:** 1 School of Health Sciences, Department of Nursing, Cyprus University of Technology, Limassol, Cyprus; 2 Birth Forward, Non-Govertmental Organization, Nicosia, Cyprus

## Abstract

**Background:**

Internet use in pregnancy is very prevalent. However, there are issues with information quality as well as acceptance by healthcare providers which can add to the frustration.

**Methods:**

An online anonymous survey, shared via Baby Buddy Cyprus, addressed women who are pregnant or recently gave birth in Cyprus. Adapting previously used questionnaires, the survey covered reasons and patterns of internet use, perceptions of trustworthiness, appraisal means and usefulness in decision-making.

**Results:**

Among 357 responses so far in this ongoing survey (38% pregnant, 62% new mums, 66% primiparas, 42% C/S, 78% private sector), searching online seems very frequent, even though 70% report coming across wrong or misleading information often. Checking for consistency across sites and/or with information by healthcare provider (HP) is the most common technique for assessing trustworthiness. While the majority discuss information with HP, only half characterize their reception as positive and welcoming. As many as 89% believe that HP should recommend sites, but only 6.5% report their HP made recommendations. The role of the internet in assisting decision-making is rated as moderate (M = 3.0, SD = 1.0 on 5-point scale averaged across 11 items); yet more than half search online to be prepared and have control over decisions. Among reasons cited for using the internet is insufficient time with HP and/or is unclear or unsatisfactory information. While only 11.6% prepare material for the next appointment, 54.5 % use the internet to verify information given by HP or for a second opinion.

**Conclusions:**

While a prevalent source of information, the flow is problematic as it appears that women are more likely to search online to verify information rather than discuss this information with their providers. Insights about characteristics and attributes of internet use in pregnancy suggest that health services need to engage with, rather than ignore, this reality and offer appropriate guidance.

**Key messages:**

• Pregnant women in Cyprus search for information online, due to insufficient time or information by healthcare providers, even though they recognize there are issues with quality and expect guidance.

• In a landscape of unguided information-seeking, searching for consistency and verification, a shift in current practices is needed whereby healthcare providers and services engage with this reality.

